# The Treatment of Acne

**Published:** 1900-09-29

**Authors:** 


### THE TREATMENT OF ACNE.

In an address delivered at Croydon on the treatment of some common diseases of the skin, Dr. Phineas S. Abraham gave some useful hints in regard to the treatment of acne, a disease which he said could be cured with certainty and in a comparatively short time by a judicious combination of internal and external remedies. The majority of the cases, he said, give some history of dyspeptic troubles, and the rosaceous cases, especially, of flushings of the face, and not infrequently among women, of the eruption being worse at the periods. Most cases then are benefited by an iron and magnesia mixture between meals, others by an alkaline bismuth mixture before food. The compound sulphur ointment of the Blackfriars Hospital should be applied to the spots every night. It contains 30 grains of sulphur, 10 grains of ammoniated mercury, 10 grains of sulphide of mercury, in an ounce of vaseline. The quantities can be varied in particular cases, and oxide of zinc may be added if there is much inflammation. Or the old sulphur and lime water lotion may be used, but the above ointment is generally the best. Before its application the patients should bathe the face with hot water and a 10 per cent. ichthyol soap well lathered on. This should be done again in the morning, and the patient may be told to keep the lather on as long as possible. A still more important part of the treatment is the application to each pimple of the tiniest droplet of pure carbolic acid, just liquified with a little water. As a rule, one such application aborts and cures the pustule, especially in recent cases of alcoholic acne. In the indurated cases where subcutaneous abscesses have formed, and where the pustules are large and deep, they should be punctured, and the cavity should be thoroughly washed out with some antiseptic lotion, such as 1 in 1,000 sublimate, 1 in 20 carbolic, izal, or chinosol. This should be done by means of a small blunt-pointed hypodermic syringe needle having the orifice at the apex. It is well also to remove as many comedones as possible, for which purpose Dr. Abraham's comedo extractor will be found useful.

